# An Ensemble Machine Learning and Data Mining Approach to Enhance Stroke Prediction

**DOI:** 10.3390/bioengineering11070672

**Published:** 2024-07-02

**Authors:** Richard Wijaya, Faisal Saeed, Parnia Samimi, Abdullah M. Albarrak, Sultan Noman Qasem

**Affiliations:** 1 College of Computing and Digital Technology, Birmingham City University, Birmingham B4 7XG, UK; richard.wijaya@mail.bcu.ac.uk (R.W.); parnia.samimi@bcu.ac.uk (P.S.); 2 Computer Science Department, College of Computer and Information Sciences, Imam Mohammad Ibn Saud Islamic University (IMSIU), Riyadh 11432, Saudi Arabia; amsbarrak@imamu.edu.sa (A.M.A.); snmohammed@imamu.edu.sa (S.N.Q.)

**Keywords:** stroke, prediction model, machine learning, ensemble learning

## Abstract

Stroke poses a significant health threat, affecting millions annually. Early and precise prediction is crucial to providing effective preventive healthcare interventions. This study applied an ensemble machine learning and data mining approach to enhance the effectiveness of stroke prediction. By employing the cross-industry standard process for data mining (CRISP-DM) methodology, various techniques, including random forest, ExtraTrees, XGBoost, artificial neural network (ANN), and genetic algorithm with ANN (GANN) were applied on two benchmark datasets to predict stroke based on several parameters, such as gender, age, various diseases, smoking status, BMI, HighCol, physical activity, hypertension, heart disease, lifestyle, and others. Due to dataset imbalance, Synthetic Minority Oversampling Technique (SMOTE) was applied to the datasets. Hyperparameter tuning optimized the models via grid search and randomized search cross-validation. The evaluation metrics included accuracy, precision, recall, F1-score, and area under the curve (AUC). The experimental results show that the ensemble ExtraTrees classifier achieved the highest accuracy (98.24%) and AUC (98.24%). Random forest also performed well, achieving 98.03% in both accuracy and AUC. Comparisons with state-of-the-art stroke prediction methods revealed that the proposed approach demonstrates superior performance, indicating its potential as a promising method for stroke prediction and offering substantial benefits to healthcare.

## 1. Introduction

In the modern era, health is the most critical aspect of every individual’s well-being. Cutting-edge smart health technologies such as the metaverse, Artificial Intelligence (AI), and data science are transforming the medical industry [1].

Stroke is a condition that occurs when there is an interruption in the blood supply to a part of the brain, leading to damage or death of brain cells. Depending on the affected area of the brain and the timeliness of treatment, stroke can result in both short-term and long-term effects. Survivors may experience a range of issues, including difficulties with mobility, speech, and cognitive and emotional functions [2]. There are two types of strokes: ischemic and hemorrhagic. The World Stroke Organization states that ischemic strokes can lead to serious diseases, such as ischemic heart disease (IHD), dementia, and Alzheimer’s disease, while hemorrhagic strokes can result in aneurysm, arteriovenous malformation (AVM), and transient ischemic attack (TIA) [2].

According to [3], there are over 100,000 stroke cases per year in the United Kingdom (UK), occurring at a rate of one every five minutes. About 1.3 million people in the UK have recovered from stroke. Researchers have used AI to build a variety of stroke prediction strategies, resulting in a huge impact, by allowing for early stroke detection and prompt patient care. Stroke is also responsible for about 75% of deaths from cerebrovascular disease. According to the Office of National Statistics (ONS), 12.7% of the deaths in the UK were caused by dementia and Alzheimer’s disease in 2018. While the total deaths from ischemic heart disease (IHD) and cerebrovascular disease have reduced over the years, IHD remains the leading cause of death among males, while dementia and Alzheimer’s disease account for 16.5% of deaths in females [4].

According to Stewart [5], the overall number of in-patients diagnosed with stroke in the UK in 2020/2021 is 128,703. Since more than 100,000 people have had a stroke every year in the past decade, this is a serious issue that needs to be overcome. Thus, predictive models are important to predict stroke disease.

Table 1 shows recent studies on the application of machine learning (ML) for predicting stroke. The outcomes of the existing studies were hindered by insufficient data or results. Therefore, this research aims to apply several ensemble machine learning methods on two benchmark datasets to find the best model that can obtain accurate and robust performance. Since class balancing is a major issue in most of the available datasets, Synthetic Minority Oversampling Technique (SMOTE) was applied. Afterwards, the methods were evaluated based on accuracy, precision, recall, F1-score, and area under the curve (AUC) values.

### 1.1. Data Analytics in Healthcare

According to the World Health Organization (WHO), there are ten leading causes of death that contributed to 55% of deaths worldwide in 2019. Among them, seven diseases were noncommunicable. The main leading cause of death was ischemic heart disease (contributed to 16% of deaths) followed by stroke (contributed to 11% of deaths) [16]. As the quantity of digital data gathered within the healthcare industry has been increasing, more technological advancements were developed to enhance medical diagnostics, early disease identification, and decision making [17].

Galetsi et al. [18] reviewed 804 studies on big data analytics (BDA) on healthcare and confirmed the significance of developing analytical techniques that offer health support and decision making by using automated algorithms. Another study [19], which performed a systematic literature review on 41 studies in healthcare, concluded that BDA could bring value to the healthcare industry in terms of conceptual evolution, data governance, decision support, disease prediction, strategy formulation, and technology development. Thus, this study focused on applying a data mining approach to stroke prediction, which can enhance decision making in the healthcare industry.

According to [20], the use of machine learning in medical imaging can be used to classify and categorize disease patterns. In the recent global pandemic, a review of medical image analysis of COVID-19 using various deep learning algorithms was conducted in [21], where convolutional neural network (CNN) was utilized to diagnose COVID-19 disease. 

In addition, the authors in [22] and [23] researched diabetic kidney failure disease by using machine learning, while the authors in [24] performed the prediction of fatty liver by using machine learning algorithms such as XGBoost, decision tree, and support vector machine. Prediction of heart disease was performed in [25] by using hybrid random forest with the linear model, which obtained 88.4% accuracy. Similar research was performed in [26], where support vector machine performed well, with 96.72% accuracy. Although there are numerous studies on different diseases, more research is required to improve the effectiveness of disease prediction.

### 1.2. Existing Studies on Stroke Prediction

In [6], predicting stroke with machine learning was investigated by using the Kaggle dataset [27]. Redundant values reduction, feature selection, data discretization, and SMOTE class balancing were performed to pre-process the data, while feature importance analysis using random forest and information gain was performed. The stacking classifier provided the best accuracy of 98% and an AUC score of 98.9%.

The authors in [10] analyzed the performance of stroke prediction by using the same dataset [27] and six different ML algorithms. Before training and evaluating each of the algorithms, data pre-processing, such as handling missing values by mean imputation, balancing imbalanced data by using undersampling, and label encoding for the categorical data, was performed on the data. Overall, Naïve Bayes performed well with an F1-score of 82.3%, an accuracy score of 82.2%, a precision score of 79.2%, and a recall score of 85.7%. Another study [11] used the same dataset [27] but with different pre-processing techniques. Missing values and label encoding were performed but without handling the imbalanced data. In addition, a weighted voting classifier was used and improved the accuracy to 97% and the AUC to 93%. 

The authors in [15] proposed different methods on the stroke prediction dataset [27] by using machine learning algorithms. The authors used principal component analysis (PCA) to find the maximum variance in the features, and ML algorithms such as neural network, decision trees, and random forests were implemented. As a result, the neural network algorithm produced the best results, with 78% accuracy and a miss rate of 19% based on a specific PCA combination feature. 

In [9], three different ML algorithms were applied. One-hot encoding was used instead of label encoding for the predictors, and several statistical tests, such as the T-test, the Wilcoxon rank sum test, and the chi-square test, were applied. Feature importance using the Gini coefficient was used. The authors used random oversampling, random undersampling, and SMOTE balancing to balance the dataset and evaluate the performance of the ML algorithms by using the 10-fold cross-validation method. As a result, the best model was random forest, with 78% accuracy and 71% AUC. The accuracy result on the unbalanced dataset was better, 95%, but the AUC value was 50%, which essentially indicated that the model with an imbalanced dataset is not able to differentiate the target variable. 

### 1.3. Potential of Machine Learning and Data Mining in Stroke Prediction

Machine learning and data mining hold significant promise in the realm of stroke prediction due to their ability to analyze huge datasets, uncover hidden patterns, and improve prediction accuracy. ML algorithms can manage and analyze large datasets that are typically found in healthcare, including patient records, medical histories, genetic information, imaging data, and more. These algorithms excel at modeling complex, non-linear relationships within the data, leading to more accurate predictions compared with traditional statistical methods [28].

One of the primary strengths of ML in stroke prediction is the ability of ensemble methods, such as random forest, ExtraTrees, and XGBoost, to enhance prediction accuracy. These techniques combine multiple models to reduce the risk of overfitting and improve generalization to new data by aggregating the results of several models [29]. Furthermore, ML models can continuously learn from new data, ensuring that predictions remain relevant and accurate over time, which is crucial in the dynamic field of healthcare, where new insights and data are constantly emerging [30].

Data mining techniques are invaluable in uncovering associations and patterns that might not be immediately apparent. For instance, they can identify correlations between lifestyle factors and stroke risk that traditional methods might miss [31]. Additionally, ML algorithms can automatically identify the most relevant features for predicting stroke, streamlining the model development process and enhancing predictive performance [32].

Another significant advantage of ML in stroke prediction is the ability to provide personalized risk assessments. By leveraging detailed patient data, ML models enable healthcare providers to tailor preventive and therapeutic interventions to individual patients, potentially improving outcomes [33]. Integrating data from various sources, such as clinical records, imaging, and genetic information, further enhances the model’s ability to make accurate predictions and offers a holistic view of patient health [34].

Numerous studies have demonstrated the effectiveness of ML in stroke prediction. Ensemble approaches like random forest and XGBoost have achieved high accuracy and area under the curve (AUC) scores in predicting stroke risk [35]. High accuracy and reliable predictions are critical in clinical settings, and ML models that consistently perform well across different datasets and patient populations are more likely to be adopted in practice [36].

The potential of machine learning has been discussed, and the existing studies have been reviewed. The main aim of this paper is to identify the best machine learning model for stroke prediction by applying data mining methodology and employing various pre-processing techniques.

## 2. Materials and Methods

**Figure 1:**
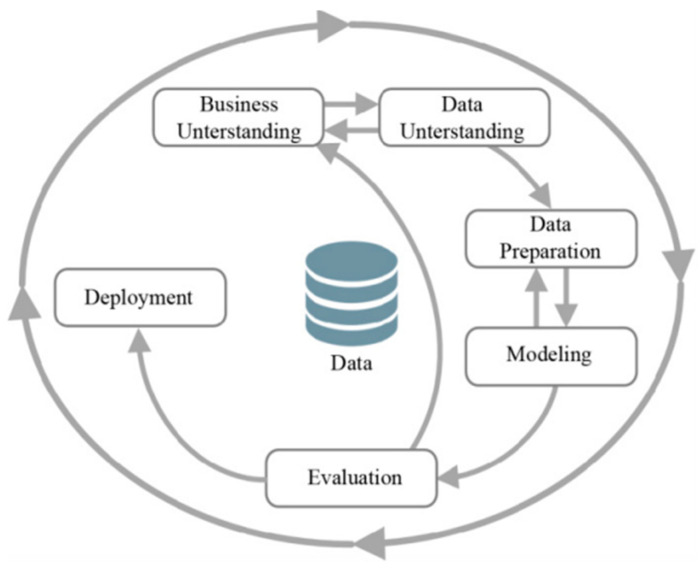
CRISP-DM methodology [37].

The cross-industry process for data mining (CRISP-DM) approach is used in this study to investigate the performance of several ensemble machine learning methods in stroke prediction (Figure 1).

### 2.1. Cross-Industry Standard Process for Data Mining (CRISP-DM) Methodology

CRISP-DM, which is used for solving several data analytics problems, is adopted as a data mining methodology. It consists of six phases: Business Understanding, Data Understanding, Data Preparation, Modeling, Evaluation, and Deployment. The literature review was conducted in the first phase to understand the problem of stroke prediction and identify the research problem and the aim of this study. The other main phases were conducted as described in the following sections.

### 2.2. Data Understanding

Two datasets were used to train and test the prediction models. The first dataset [27] includes 12 features, such as gender, age, and previous medical records which might be associated with a patient’s likelihood of getting a stroke. The attributes of this dataset are detailed in Table 2.

The second dataset [38] includes slightly different features, such as high cholesterol, cholesterol check, physical activity, and others. The details of these features are shown in Table 3.

### 2.3. Data Preparation

To gain more understanding about the datasets, different data preparation methods were adopted, such as feature selection, feature importance, handling missing data, data balancing, data splitting, and data normalization. For instance, feature selection was conducted to remove the variables that are not significant for stroke prediction, such as the ‘ID’ variable. Moreover, feature importance using the chi-square test was used to determine variable importance for modeling.

Feature importance analysis is crucial when working with stroke prediction, as it identifies key predictors within the dataset, enhancing model performance. By highlighting the most important features, it guides clinical decision making, helping healthcare providers focus on relevant factors and improve diagnostic accuracy. This analysis also aids in refining models for better performance and efficiency and supports personalized medicine by aiding in preparing treatment plans individual patients.

Python library ‘pandas’ was used to perform data pre-processing. The first step is to identify the missing values. For instance, 202 missing values were found in the first dataset and were removed, as the dataset has sufficient data for model training.

Normalization was performed to execute data transformation, converting categorical data into numerical data by using label encoding and one-hot encoding. The main difference between the two techniques is that label encoding is performed when the data type is ordinal and the hierarchy within that attribute matters, whereas one-hot encoding is applied if ordinal data do not exist. Additionally, data balancing of the class variable ‘stroke’ in both datasets was performed by using Synthetic Minority Oversampling Technique (SMOTE), which is an oversampling method to prevent bias or skewness within the stroke prediction model which would result in an inaccurate prediction.

SMOTE is a method used to address class imbalance in datasets. It works by generating synthetic examples for the minority class to balance the class distribution. This is achieved by taking each minority class sample and introducing synthetic examples along the line segments joining the k minority class nearest neighbors. This technique helps in improving the performance of classifiers on imbalanced datasets by providing more representative samples of the minority class [39].

Lastly, data splitting was performed, where the datasets were divided into training and testing data in an 80:20 ratio. In addition, both the minmax and standard scalers were applied to the input training dataset to compare the performance of different machine learning algorithm on the original data without feature scaling, on the data after application of the minmax scaler, and the data with after application of the standard scaler.

### 2.4. Modeling

In this paper, several ensemble machine learning methods were trained and tested, which include random forest, Gradient Boost, histogram-based Gradient Boosting, XGBoost, LightGBM, CatBoost, and ExtraTrees. In addition, artificial neural network (ANN) with genetic algorithm was applied to train the prediction model. 

Boosting algorithms such as AdaBoost, Gradient Boosting, XGBoost, LightGBM, and CatBoost, alongside histogram-based Gradient Boosting, are pivotal in reducing bias and variance in models [40]. AdaBoost (Adaptive Boosting) enhances the performance of any given learning algorithm by iteratively adjusting the weights of incorrectly classified instances, thus focusing more on hard-to-predict cases in subsequent iterations. This method helps reduce bias by improving the accuracy of weak classifiers and transforming them into strong ones [40].

Gradient Boosting, on the other hand, builds models sequentially, with each new model correcting the residual errors of the previous ones. This approach effectively reduces bias and variance by optimizing the loss function during the learning process, resulting in a strong predictive model. Regularization techniques in Gradient Boosting further help in mitigating overfitting, making the model robust against noise in the training data [41].

XGBoost, an advanced implementation of Gradient Boosting, integrates several enhancements, such as regularization parameters to control model complexity, tree pruning to eliminate unnecessary splits, and parallel processing for efficient computation. These features make XGBoost particularly effective in achieving high prediction accuracy while maintaining a balance between bias and variance, making it a powerful tool for stroke prediction [42]. Similarly, LightGBM and CatBoost integrate optimizations like histogram-based algorithms, which accelerate training and enhance performance in large datasets, further contributing to bias–variance trade-off management [43,44]. Furthermore, histogram-based Gradient Boosting techniques efficiently process data in histogram bins, reducing computation time and improving model efficiency [42].

In addition to boosting algorithms, other techniques, such as artificial neural network (ANN) and genetic algorithm (GA), also contribute to bias–variance management. ANN, with its ability to capture complex patterns in data, offers another approach to reducing bias and variance [32]. Genetic algorithm, when used in conjunction with ANN, aids in optimizing network architecture and hyperparameters, further enhancing model performance [45]. By iteratively optimizing the architecture and parameters of ANN by using GA, this approach maximizes the model’s ability to capture relevant patterns in the data while avoiding overfitting [45]. Consequently, the combination of GA with ANN provides a powerful framework for managing bias and variance in predictive modeling tasks.

### 2.5. Evaluation

Evaluation metrics include accuracy, precision, recall, F1-score, and area under the curve (AUC), which were used to assess the model performance.

True Positive (TP) reflects the values where both actual and predicted values are true whereas false negative (FN) reflects values where both actual and predicted are false. False Positive (FP) reveals where the actual value is false, and the predicted value is true, whereas True Negative (TN) reflects values where both the actual and predicted outcomes are negative. The evaluation metric formulas are shown in Table 4.

The area under the curve (AUC) plots the True Positive Rate (sensitivity) and False Positive Rate (1—specificity). The AUC is one of the evaluation metrics for binary classification problems, as it represents the ability of the model to differentiate the target classes. Therefore, a high AUC value indicates that the model can distinguish between the positive and negative classes, while AUC values that are near zero indicate that the model is detecting negative classes as positive ones and vice versa. Lastly, in the case where the AUC value is equal to 0.5, the model is unable to categorize the target classes, which essentially makes the model redundant.

Upon completion of model training, the performance of the models was compared to that of current state-of-the-art stroke prediction models to assess if the proposed model made a significant advancement for the medical field.

### 2.6. The Proposed Approach

**Figure 2:**
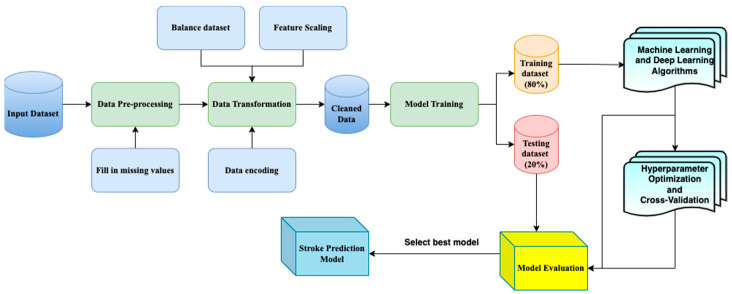
Flowchart of the methodology.

Figure 2 presents the approach proposed in this study. As presented above, the two datasets were pre-processed to remove data duplication, missing values, and inconsistent data. Additionally, variable selection and data transformation were performed to prepare the datasets for model training. The datasets were then separated in 80:20 ratios, and several machine learning methods, including random forest, ExtraTrees, XGBoost, artificial neural networks (ANN), and genetic algorithm with ANN (GANN) were applied. Upon completion of model training, hyperparameter optimization and cross-validation were implemented. Finally, each of the models was evaluated based on its performance on the two datasets, and the model with the highest performance was reported as the best stroke prediction model. In addition, a comparison of the best-performing model with current state-of-the-art stroke prediction models was undertaken.

## 3. Experimental Results

To analyze and understand the datasets well before applying the machine learning methods, Figure 3, Figure 4, Figure 5, Figure 6, Figure 7, Figure 8, Figure 9, Figure 10 and Figure 11 show the visualization of the first dataset, while Figure 12, Figure 13, Figure 14, Figure 15, Figure 16, Figure 17, Figure 18 and Figure 19 show the visualization of the second dataset.

**Figure 3:**
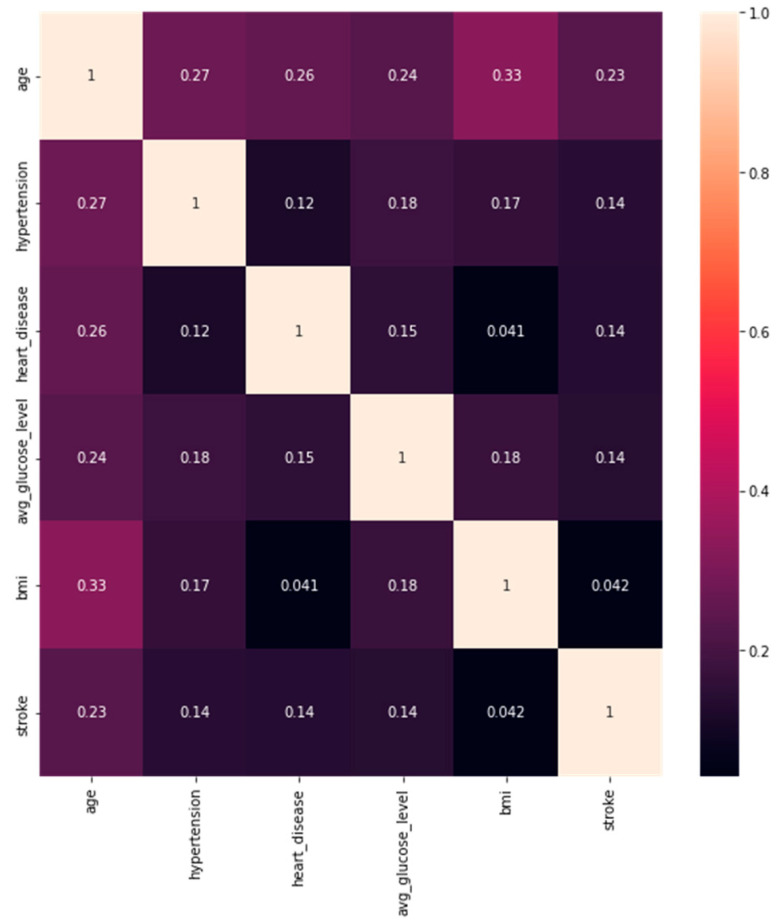
Correlation plot.

Figure 3 shows the correlation between the numerical variables within the first dataset. There are six numerical variables, and none of them shows a strong correlation with the others. However, a weak correlation of 0.33 was identified between the variables age and BMI. Another weak correlation of 0.27 was identified between hypertension and age. The target variable, ‘stroke’, has no strong correlation with the other input variables.

**Figure 4:**
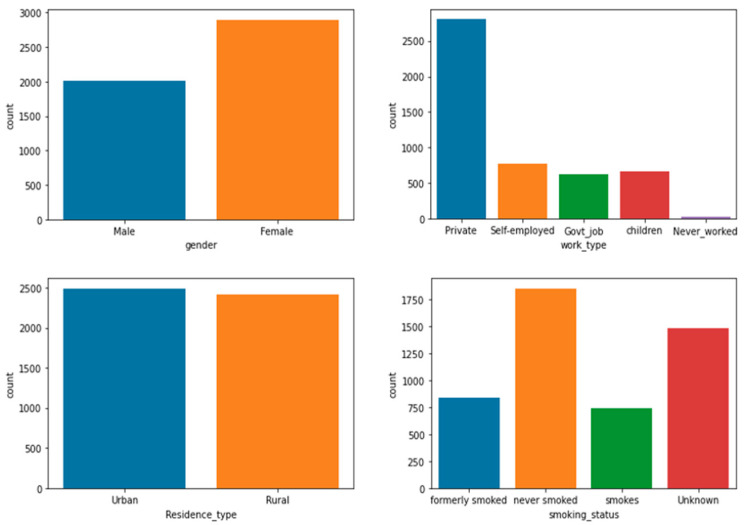
Count plot for attributes ‘gender’, ‘work type’, ‘residence type’, and ‘smoking status’.

Figure 4 describes four different count plots for the categorical variables in the first dataset. Firstly, the ‘gender’ variable shows the patient demographics of the dataset, where approximately 3000 patients were female and the remaining 2000 were male. Thus, 60% of the patients were female, whereas the remaining 40% were male patients. In terms of ‘work type’, 57% of the patients worked in the private sector, with about 2800 patients, and a similar number of patients were in the categories self-employed, government-based jobs, or children. However, a very small percentage of the patients had never worked before. Regarding the place of residency, 50% of the patients lived in an urban area, whereas the other half lived in a rural area. Lastly, 38% of the patients had never smoked before, 15% of the patients were active smokers, 17% of the patients had smoked before, and the remaining 30% had an unknown history of smoking.

**Figure 5:**
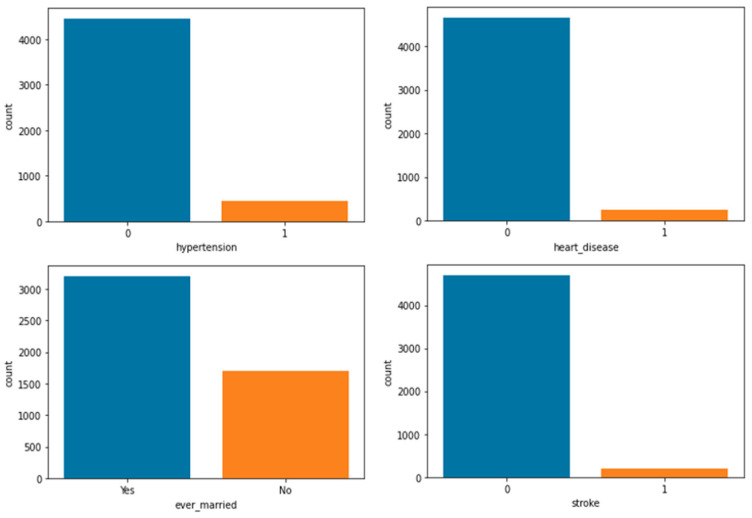
Count plot for attributes ‘hypertension’, ‘heart disease’, ‘ever married’, and ‘stroke’.

Figure 5 illustrates the remaining four different count plots for the binary variables in the first dataset. Firstly, 65% of the patients had no history of hypertension, whereas 35% of the patients had hypertension. In terms of heart disease, 95% of the patients had no history of heart disease, while the remaining 5% of the patients had heart disease. Similarly, 95% of the patients were married. Lastly, for the target variable ‘stroke’, 95% of the patients in the first dataset had not had a stroke, which means that the dataset is imbalanced. Thus, a class balancing technique called SMOTE was implemented during data transformation.

**Figure 6:**
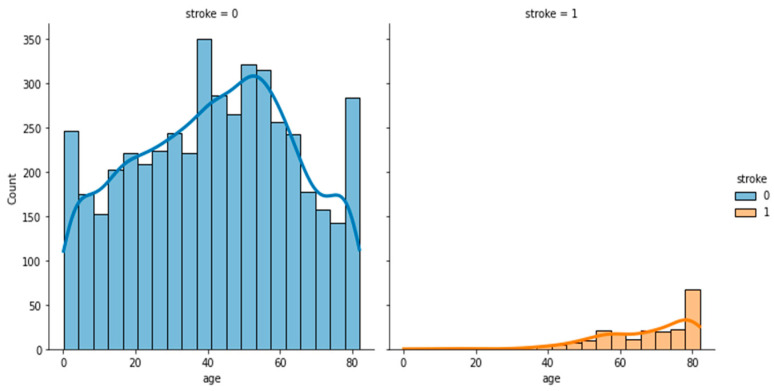
Distribution plot of age based on stroke.

**Figure 7:**
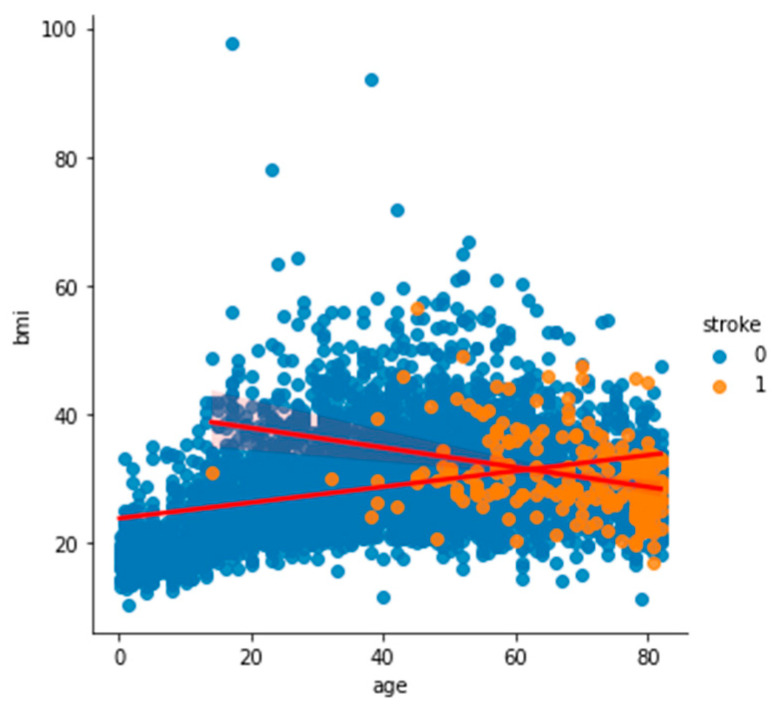
Scatter plot of age against BMI based on stroke.

Figure 6 shows the histogram of the age variable categorized based on the target variable ‘stroke’. Most of the patients who had had a stroke were above the age of 40. Figure 7 also shows that people aged over 40 with a BMI of around 30 had had a stroke.

**Figure 8:**
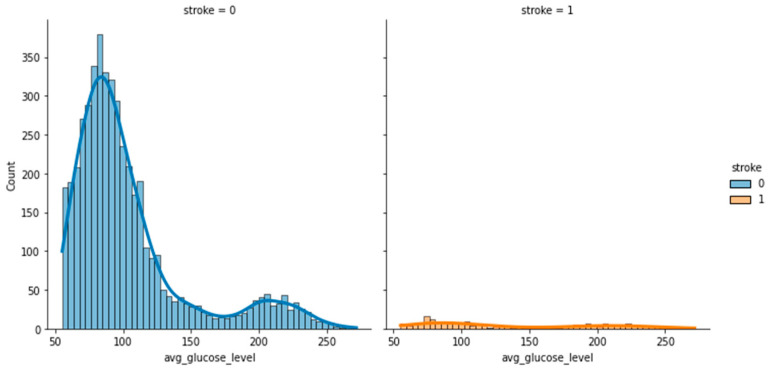
Distribution plot of average glucose level based on Stroke.

**Figure 9:**
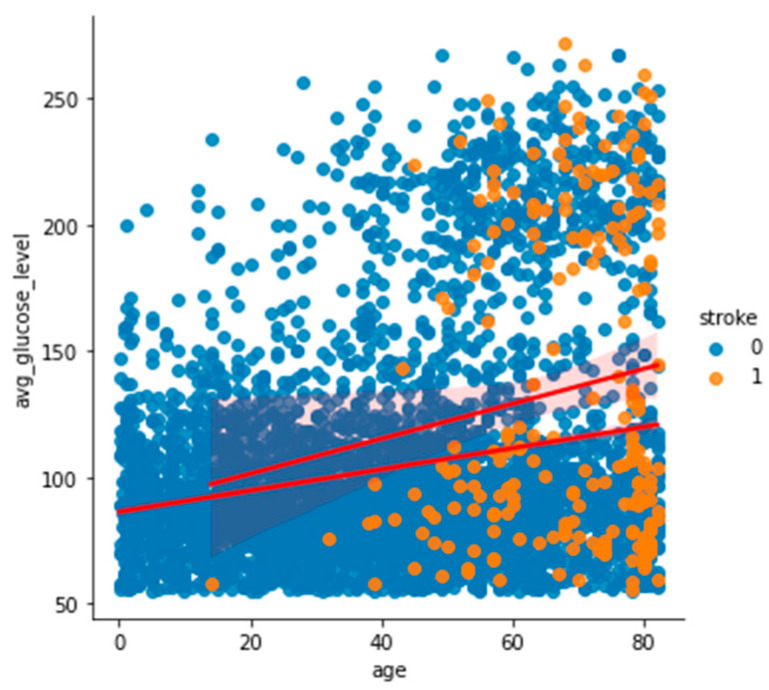
Scatter plot of age against average glucose level based on stroke.

Figure 8 shows the histogram of the average glucose level categorized based on the target variable ‘stroke’. It indicates that patients who had had a stroke had a low average glucose level of 90–110. A similar trend is also seen in Figure 9, where patients who had had a stroke had an average glucose level of less than 100.

**Figure 10:**
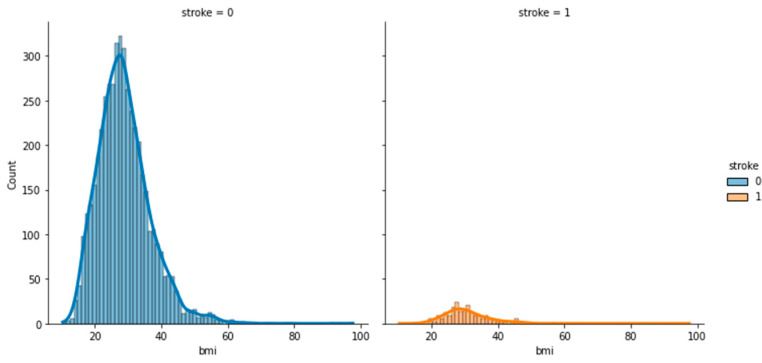
Distribution plot of BMI based on stroke.

**Figure 11:**
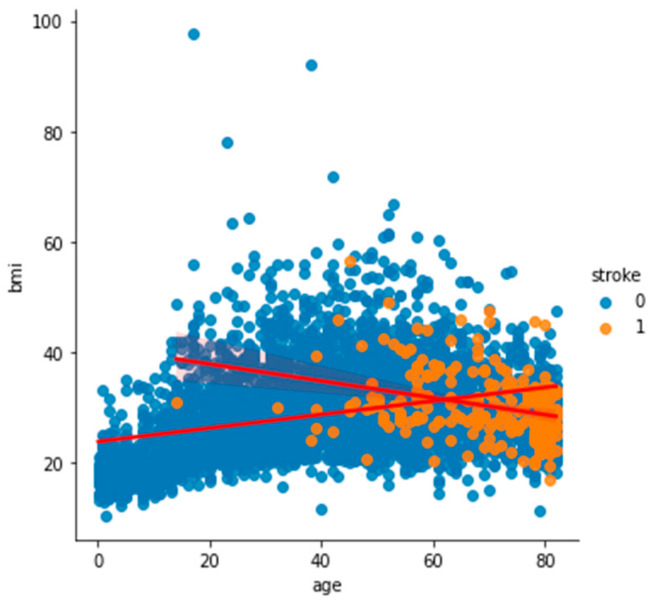
Scatter plot of age against BMI based on stroke.

Figure 10 shows the histogram of BMI based on stroke, which indicates that most patients who had suffered from stroke were in the range of 30–35. Similarly, in Figure 11, patients who had had a stroke had the BMI of an overweight person, as a normal BMI is between 18.5 and 24.9. In summary, patients with these characteristics are at a higher risk of experiencing a stroke: being overweight, having low average glucose levels, and being above the age of 40.

**Figure 12:**
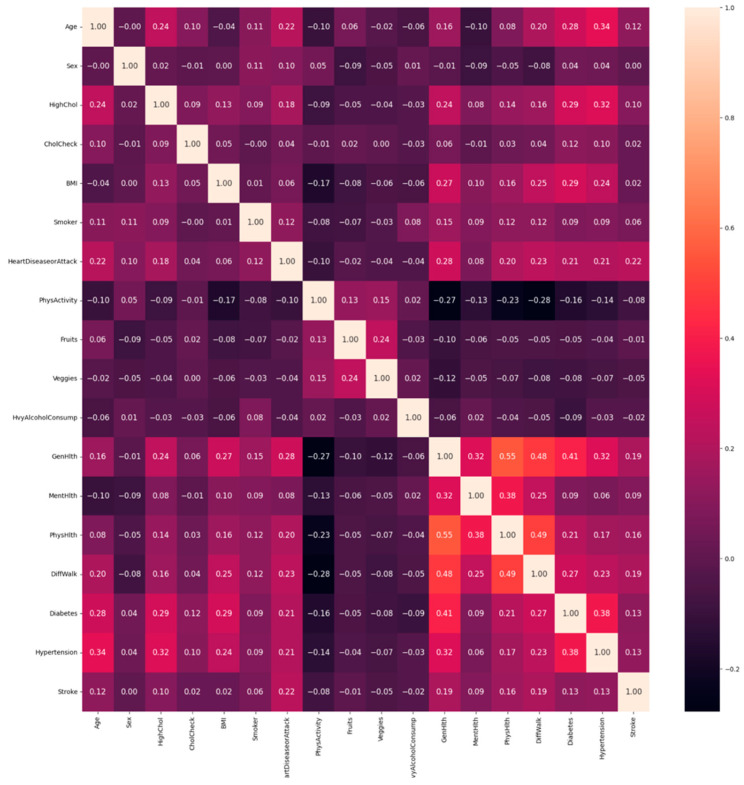
Correlation plot.

Figure 12 shows the correlation between the numerical variables only within the second dataset. Unlike the first dataset, there are moderately correlated variables. The variables ‘physical health’ and ‘general health’ have a moderate correlation of 0.55 followed by a correlation between ‘different walk’ and ‘physical health’ of 0.49, as well as one between ‘general health’ and ‘different walk’ of 0.48. Similar to the first dataset, the target variable ‘stroke’ is not strongly correlated with any of the input variables.

**Figure 13:**
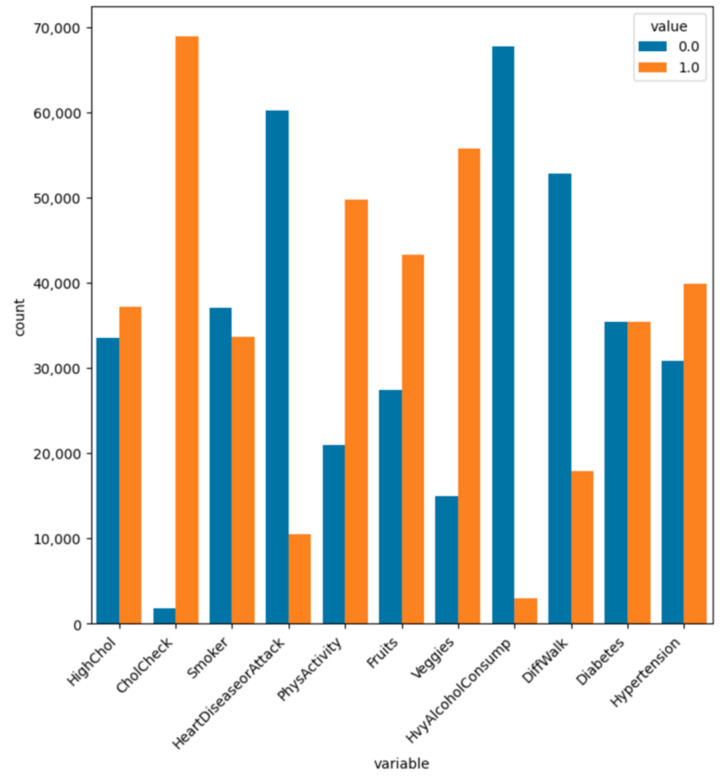
Count plot for attributes ‘high cholesterol’, ‘cholesterol check’, ‘smoker’, ‘heart disease or attack’, ‘physical activity’, ‘fruits’, ‘veggies’, ‘heavy alcohol consumption’, ‘different walk’, ‘diabetes’, and ‘hypertension’.

Figure 13 shows the count plot for all the binary variables in the second dataset. Firstly, 52% of the patients had high cholesterol, and 97% of the patients had performed at least one cholesterol check in the past 5 years. In terms of smoking, 52% of the patients had never smoked, and 85% of the patients did not have heart disease or had not experienced a heart attack. Additionally, 70% of the patients had performed physical activities in the past month. Regarding fruit and vegetable consumption, 61% and 78% of the patients ate one or more fruits and vegetables every day, respectively, and 95% of the patients did not consume heavy alcohol; further, 74% of the patients had not had difficulty in walking in the past month. However, 50% of the patients suffered from diabetes despite their good eating habits and exercise, and 56% of the patients suffered from hypertension.

**Figure 14:**
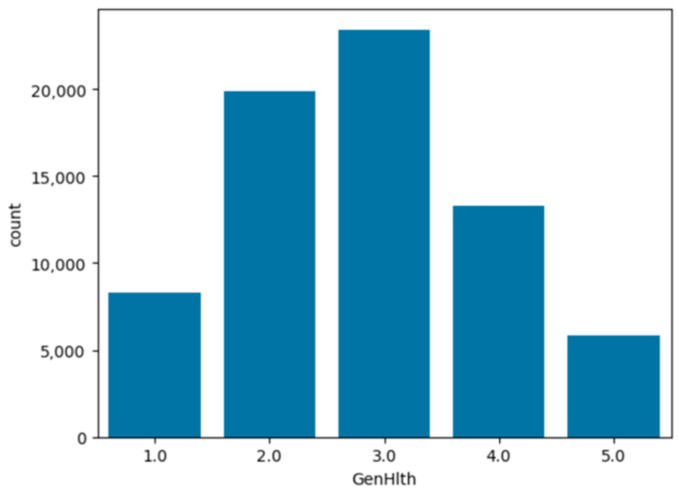
Count plot on ‘general health’ variable.

Figure 14 describes the patients’ general health for the past month on a scale of 1 to 5 where 1 is excellent and 5 is poor. A total of 80% of the patient population experienced an excellent to average feeling of their general health for the past month, while the remaining 20% had poor feelings regarding their general health for the past month.

**Figure 15:**
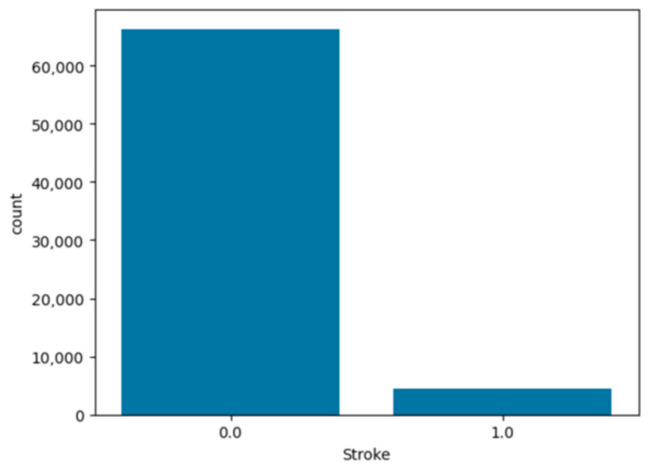
Count plot for ‘stroke’ variable (target variable).

Figure 15 shows the count plot for the target variable ‘stroke’ and clearly shows that the target variable is not balanced, where 93% of the patients had not been diagnosed with stroke. Similar to the first dataset, since the target variable is not balanced, the SMOTE technique was applied to balance the dataset.

**Figure 16:**
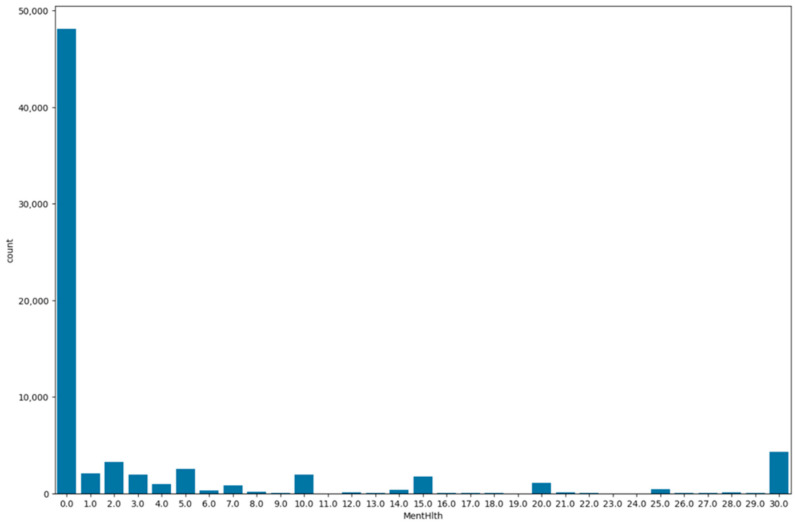
Bar plot for mental health variable.

The variable ‘mental health’ is visualized on a bar plot as shown in Figure 16, indicating the number of days on which the patients had poor mental health. A total of 68% of the patients did not feel that they had a poor mental health day, and 10% of the patients felt like they had had 1 to 5 days of poor mental health. On the other hand, 6% of the patients felt like every day for the past 30 days, they had poor mental health. 

**Figure 17:**
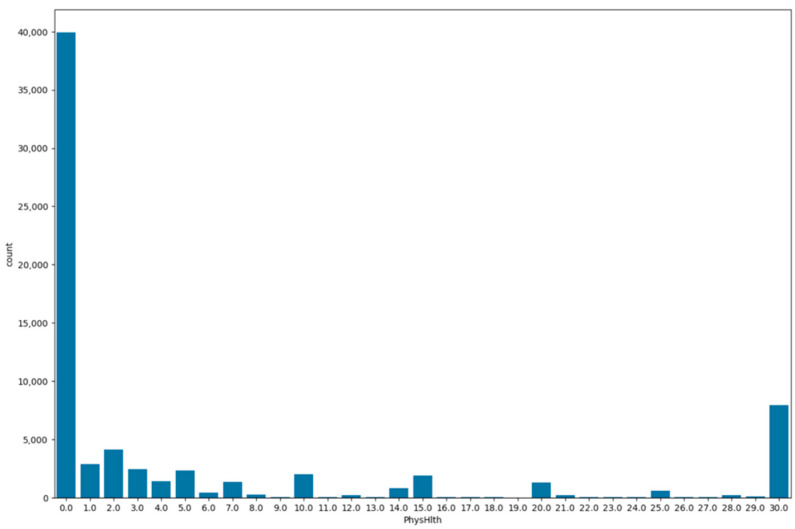
Bar plot for physical health variable.

Similarly, the variable ‘physical health’ is visualized on a bar plot as shown in Figure 17, indicating the number of days on which the patients experienced physical illness or injuries in the past month. A total of 56% of the patients were healthy, followed by 11% of the patients that experienced physical injuries or illness on all the past 30 days. On the other hand, 18% of the patients had 1 to 5 days of physical injuries or illness.

**Figure 18:**
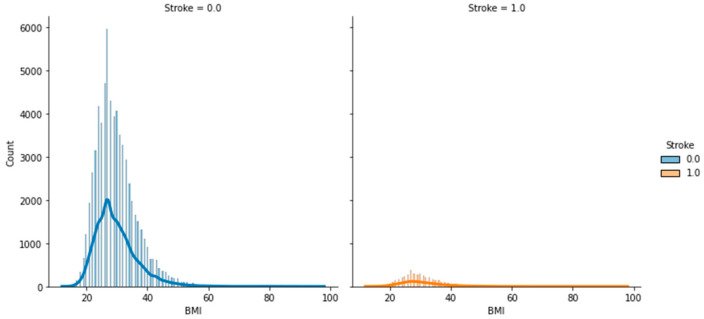
Distribution plot of BMI based on stroke.

**Figure 19:**
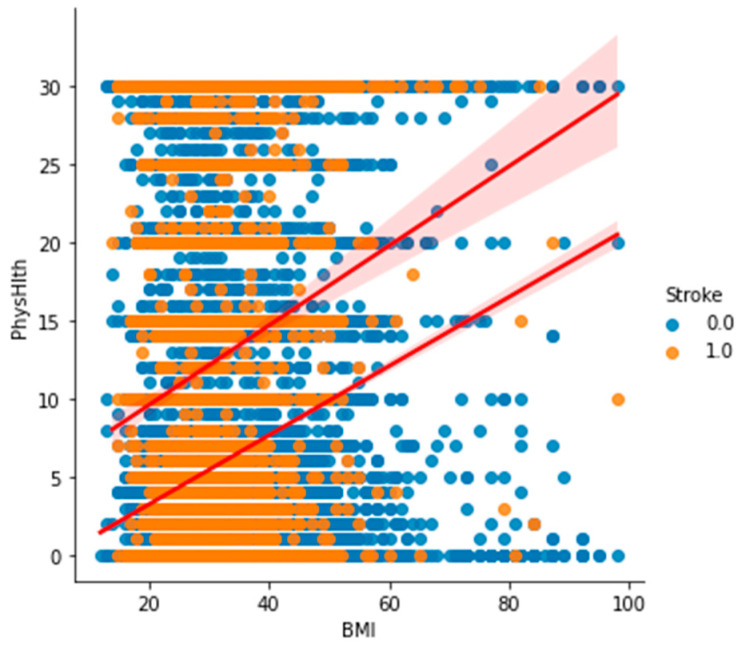
Scatter plot of BMI against physical health based on stroke.

Figure 18 shows the histogram of BMI based on stroke, which indicates that patients who had had stroke had a BMI above average (30). Similarly, in Figure 19, which is a scatter plot based on BMI against physical health based on stroke, many patients had had a stroke when their BMI was overweight. Additionally, the patient that had less injury and illness physically also had had a stroke.

**Figure 20:**
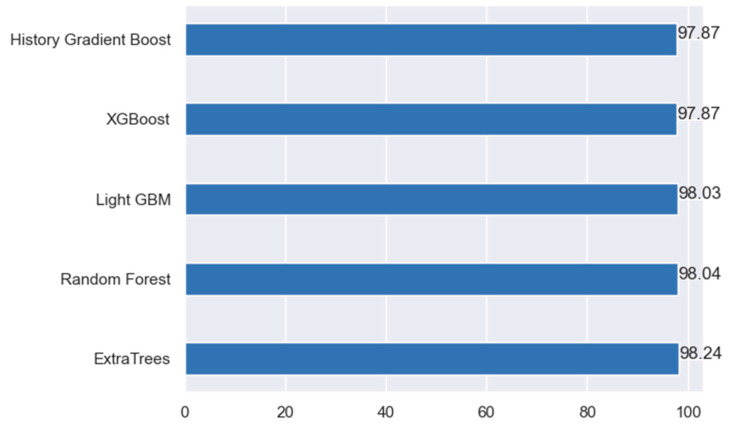
Comparison of top 5 performing models (accuracy) on the first dataset.

Figure 20 shows the top five machine learning models, based on the accuracy on the first dataset. Both light GBM and random forest algorithms performed well and delivered high accuracy. However, the best-performing model was the ExtraTrees classifier.

**Figure 21:**
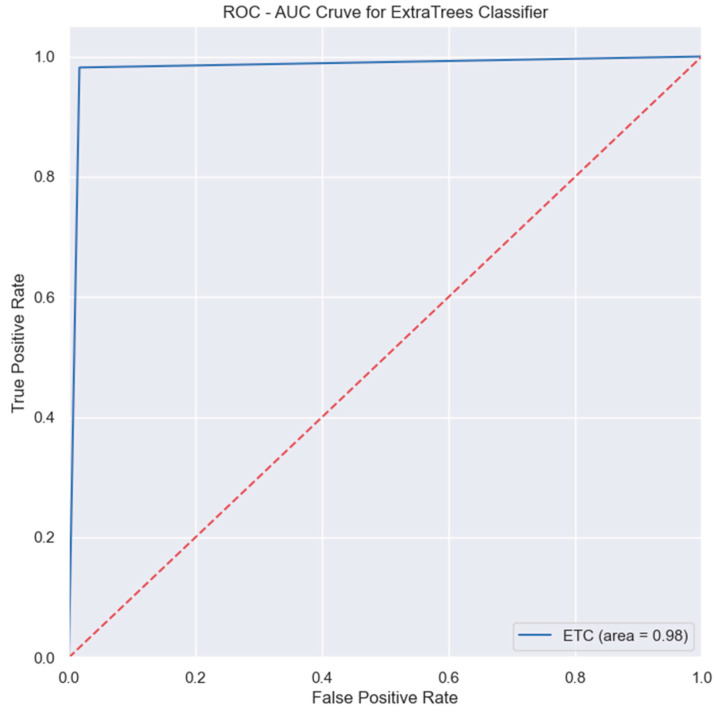
ROC-AUC graph of ExtraTrees classifier.

Figure 21 shows that the AUC value of the ExtraTrees classifier is 0.98, which indicates that the model can differentiate the target class distinctively. The full results of the model performance on the first dataset are shown in Table 5. Here, we can see that although the GANN algorithm without feature selection and class balancing is not listed in the top five performing models, this model obtained an accuracy of 96%. However, the target variable is not balanced, which creates a biased prediction model.

**Figure 22:**
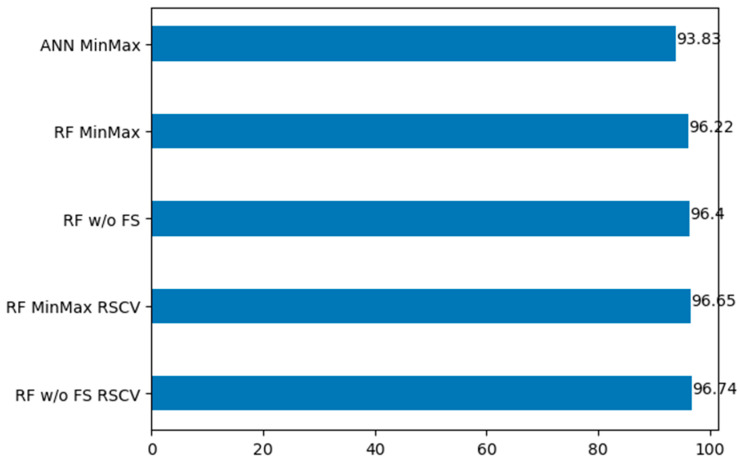
Comparison of the top 5 performing models (accuracy) on the second dataset.

Figure 22 shows the top five models based on their accuracy on the second dataset. It shows that applying the standard scaler on random forest on the second dataset reduces the model performance significantly, while applying the minmax scaler on random forest produces a better result. As in the first dataset, the ANN algorithm is shown to be suitable for stroke prediction on the second dataset, achieving an average accuracy of 92%. However, the best-performing model on the second dataset is random forest without feature scaling and applying randomized cross-validation, with 96.74% accuracy. Additionally, as seen in Table 3, the AUC of the model is 0.97, which indicates that the model can differentiate the target class distinctively. However, the GANN algorithm is not listed in the top five, which indicates that it is not suitable for stroke prediction. The results of the performance of the applied models on the second dataset are presented in Table 6.

## 4. Discussion

Table 7 compares the proposed model with the state-of-the-art stroke prediction models. In this comparison, it shows that most of the previous studies used the Stroke Prediction dataset [27]. Other than that, different evaluation metrics were used in these studies. Therefore, the comparison was performed according to the available results based on different data pre-processing techniques.

It is shown that the ExtraTrees classifier is the best-performing model, as it achieved an accuracy of 98.24%; despite the SVM model by [8] having a better accuracy of 99.99%, the author did not provide the AUC score, which is an important aspect when evaluating a model. The authors in [8,10,11,15] used the same dataset, but their models did not produce significant results, as they used different methods of data pre-processing and transformation.

Compared with the other studies, the research works by [7,9] used different datasets, and their best-performing model is random forest in both cases, achieving 97.62% and 78% accuracy, respectively. However, the proposed model achieved better overall evaluation metrics and AUC values.

In terms of AUC, the proposed model achieved an AUC of 98%, indicating strong discrimination abilities, while the weighted voting algorithm in [11] and random forest algorithm in [9] had significant AUC scores of 93% and 71%, respectively.

Overall, it is shown that ensemble machine learning models such as ExtraTrees and random forest algorithm perform well in stroke prediction, achieving particularly high accuracy and AUC scores. Cross-validation techniques were applied to prevent overfitting and validate the data. In addition, ANN and GANN were applied and compared with the ExtraTrees classifier model.

However, complex ensemble models, while they often produce accurate results, can lack interpretability. Understanding how these models make predictions is crucial, especially in sensitive domains like healthcare. Challenges in interpretability include the black-box nature of many ensemble models, as well as their complexity arising from the combination of multiple base learners. Additionally, the non-linear relationships captured by ensemble models can further complicate interpretation. To address these challenges and ensure transparency and explainability, several techniques can be employed. Feature importance analysis calculates feature importance scores, indicating how much each feature contributes to the model’s predictions.

Integrating multi-modal data, such as clinical records, imaging data, and genetic information, is crucial to enhancing predictive accuracy and facilitating personalized treatment plans for stroke patients. Each data modality provides unique insights into different aspects of stroke risk and patient health status.

## 5. Conclusions

This research aimed to predict the risk of stroke at an early stage, which can help to reduce the death rate and increase the survivor rate in stroke patients. The high number of stroke cases in the UK in recent years highlights the importance of developing effective predictive models. This research aimed to develop a predictive classification model for the occurrence of stroke and assess the performance of the model by using appropriate evaluation metrics. Additionally, a data mining methodology was used. The findings indicate that ensemble methods, particularly the ExtraTrees classifier, achieved the highest accuracy, 98.24%, and the highest area under the curve (AUC), also 98.24%. This performance is superior to that of other models, including random forest, which also performed well, with 98.03% accuracy and AUC. These results highlight the effectiveness of ensemble methods for stroke prediction. Data pre-processing played a crucial role in improving model performance. The use of Synthetic Minority Oversampling Technique (SMOTE) for handling class imbalance, along with normalization and feature selection, was essential. These pre-processing steps ensured that the models were trained on balanced and well-prepared data, leading to more reliable predictions. When compared with the existing state-of-the-art stroke prediction methods, the proposed ensemble model demonstrated superior performance, indicating its potential for clinical application. This finding underscores the effectiveness of the advanced machine learning techniques employed in the study.

The integration of various data types, including demographics, medical history, and lifestyle factors, was emphasized as essential to enhancing predictive accuracy and providing personalized treatment plans. This multi-modal data integration ensures that predictions are comprehensive and tailored to individual patients, improving the overall healthcare outcome. While ensemble methods like ExtraTrees and random forest offer high accuracy, this study acknowledges the challenge of interpretability in complex models. To address this, techniques such as feature importance analysis are proposed, ensuring that the models remain transparent and explainable. This balance between high performance and interpretability is crucial for the adoption of these models in clinical settings.

## Figures and Tables

**Table 1:** Recent studies for stroke prediction.

Author	Best Model (Accuracy)
[6]	Stacking: 97.4%
[7]	Random forest: 97.6%
[8]	SVM: 99.9%
[9]	Random forest: 78.0%
[10]	Naïve Bayes: 82.0%
[11]	Weighted voting: 97.0%
[12]	DNN: 84.0%
[13]	CNN-bi-LSTM: 94%
[14]	ANN: 95.3%
[15]	Neural network: 77.0%

**Table 2:** Stroke dataset description.

Attribute Name	Data Type	Description
ID	Numeric	Unique identifier of each patient
Gender	Categorical	Gender of the patient
Age	Numeric	Age of the patient
Hypertension	Numeric	0 means that the patient does not have hypertension 1 means that the patient has hypertension
Heart disease	Numeric	0 indicates the patient does not have heart disease 1 indicates that the patient has heart disease
Ever married	Categorical	“Yes” means that the patient is married “No” means that the patient is not married
Work type	Categorical	The work that each patient does, categorized into ‘children’, ‘Govt_Job’, ‘Never Worked’, and ‘Self-employed’
Residence type	Categorical	The residence type of each patient is categorized into rural or urban area
Average glucose level	Numeric	The blood glucose level of the patient
BMI (Body Mass Index)	Numeric	The BMI of each patient
Smoking status	Categorical	The smoking status of patients is categorized into ‘Formerly smoked’, ‘Never smoked’, ‘Smokes’, or ‘Unknown’
Stroke	Numeric	The target variable indicates whether the patient has had a stroke or not

**Table 3:** The second stroke prediction dataset description.

Attribute Name	Data Type	Description
Age	Numeric	Age of the patient in 13 categories
Sex	Numeric	Patient gender, where (1) is male and (0) is female
HighChol	Numeric	Cholesterol in the patient: (0) not high cholesterol and (1) cholesterol is high
CholCheck	Numeric	Cholesterol in the patient, where (0) indicates no cholesterol check for the past 5 years and (1) means that there have been cholesterol checks in the past 5 years
BMI	Numeric	Body Mass Index
Smoker	Numeric	This binary variable indicates whether the patient has smoked more than 100 cigarettes in their entire life
HeartDiseaseorAttack	Numeric	This is a binary variable which indicates whether the patient has a history of coronary heart disease (CHD) or not
PhysActivity	Numeric	This variable represents whether the patient has performed any physical activity for the past month, where (0) is no and (1) is yes
Fruits	Numeric	This binary variable indicates whether the patient consumes one or more fruits per day
Veggies	Numeric	This binary variable indicates whether the patient consumes one or more vegetables per day
HvyAlcoholConsump	Numeric	This binary variable represents heavy alcohol consumption of more than 14 drinks per week for men and more than 7 drinks for women.
GenHlth	Numeric	This binary variable represents the patient’s general health on a scale of 1 to 5, where 1 is excellent and 5 is poor

**Table 4:** Confusion matrix formula.

Metrics	Formula
Accuracy	$\frac{TP+TN}{TP+TN+FP+FN}$
Precision	$\frac{TP}{TP+FP}$
Recall (sensitivity)	$\frac{TP}{TP+TN}$
F1-score	$2\times \frac{Precision\times Recall}{Precision+Recall}$
Specificity	$\frac{TN}{FP+TN}$

**Table 5:** Model results on the first dataset.

Model	Evaluation Metrics				
	Accuracy	Precision	Recall	F1-Score	AUC
Gradient Boost	97.29%	97.38%	97.29%	97.29%	97%
Histogram-based Gradient Boosting	97.87%	97.89%	97.87%	97.87%	98%
XGBoost	97.87%	97.88%	97.87%	97.87%	98%
LightGBM	98.03%	98.05%	98.03%	98.03%	98%
CatBoost	97.45%	97.50%	97.45%	97.45%	97%
ExtraTrees Classifier	**98.24%**	**98.24%**	**98.24%**	**98.24%**	**98%**
Random forest	98.03%	98.05%	98.03%	98.03%	98%
Random forest without feature scaling and class balancing	94.50%	89.49%	94.50%	91.93%	50%
Random forest with minmax scaler	96.65%	96.72%	96.65%	96.65%	97%
Random forest with standard scaler	85.32%	87.69%	85.32%	85.04%	85%
Random forest without feature scaling + RSCV	97.18%	97.26%	97.18%	97.18%	97%
Random forest with minmax scaler + RSCV	97.29%	97.38%	97.29%	97.29%	97%
Random forest with standard scaler + RSCV	77.77%	84.20%	77.77%	76.55%	77%
ANN without feature scaling and class balancing	94.60%	89.50%	94.60%	91.98%	83%
ANN without feature scaling	92.13%	92.14%	92.13%	92.13%	97%
ANN with minmax scaler	94.89%	94.89%	94.89%	94.89%	99%
ANN with standard scaler	95.43%	95.43%	95.43%	95.43%	99%
ANN without feature scaling + GSCV	87.93%	88.50%	87.93%	87.86%	94%
ANN with minmax scaler + GSCV	95.96%	96.05%	95.96%	95.96%	99%
ANN with standard scaler + GSCV	96.33%	96.38%	96.33%	96.33%	99%
GANN without feature scaling and class balancing	96.03%	-	-	-	-
GANN without feature scaling	75.67%	-	-	-	-
GANN with minmax scaler	76.79%	-	-	-	-
GANN with standard scaler	79.98%	-	-	-	-

**Table 6:** Model results on the second dataset.

Model	Evaluation Metrics				
	Accuracy	Precision	Recall	F1-Score	AUC
Random forest without feature scaling and class balancing	92.96%	89.27%	92.96%	90.67%	52%
Random forest without feature scaling	96.40%	96.50%	96.40%	96.40%	96%
Random forest with minmax scaler	96.22%	96.32%	96.22%	96.21%	96%
Random forest with standard scaler	49.61%	62.95%	49.61%	33.07%	50%
Random forest without feature scaling + RSCV	96.74%	96.88%	96.74%	96.73%	97%
Random forest with minmax scaler + RSCV	96.65%	96.79%	96.65%	96.64%	97%
Random forest with standard scaler + RSCV	49.53%	62.38%	49.53%	32.84%	50%
ANN without feature scaling and class balancing	93.65%	87.70%	93.65%	90.58%	81%
ANN without feature scaling	89.38%	89.50%	89.38%	89.37%	96%
ANN with minmax scaler	93.83%	94.01%	93.83%	93.82%	98%
ANN with standard scaler	92.08%	92.33%	92.08%	92.07%	98%
ANN without feature scaling + GSCV	91.27%	91.44%	91.27%	91.26%	97%
ANN with minmax scaler + GSCV	93.17%	93.61%	93.17%	93.15%	98%
ANN with standard scaler + GSCV	91.04%	91.06%	91.04%	91.04%	97%
GANN without feature scaling and class balancing	93.82%	-	-	-	-
GANN without feature scaling	72.27%	-	-	-	-
GANN with minmax scaler	76.01%	-	-	-	-
GANN with standard scaler	73.21%	-	-	-	-

**Table 7:** Comparison of proposed model and the current state-of-the-art stroke prediction models.

Author	Best Algorithm	Data Pre-Processing	Dataset	Accuracy	AUC
The proposed model	ExtraTrees Classifier	SMOTE balancing, One-hot encoding, and label encoding	Stroke Prediction Dataset by [27]	98.24%	98%
[7]	Random forest	Normalization and agglomerative hierarchal clustering	Open-Source Healthcare Dataset Stroke Data	97.62%	81%
[8]	SVM	Class balancing and hyperparameter optimization	Stroke Prediction Dataset by [27]	99.99%	-
[9]	Random forest	SMOTE balancing	Chinese Longitudinal Healthy Longevity Study (CLHLS) dataset for stroke prediction from 2012 and 2014	78.0%	71%
[10]	Naïve Bayes	Undersampling class balancing	Stroke Prediction Dataset by [27]	82.0%	-
[11]	Weighted voting	Data normalization	Stroke Prediction Dataset by [27]	97.0%	93%
[15]	Neural network	Principal component analysis	Stroke Prediction Dataset by [27]	77.0%	-

## Data Availability

The datasets are available online at [27,38].
